# Association between work-related physical activity and depressive symptoms in Korean workers: data from the Korea national health and nutrition examination survey 2014, 2016, 2018, and 2020

**DOI:** 10.1186/s12889-023-16631-6

**Published:** 2023-09-08

**Authors:** Min Jeong Joo, Ye Seul Jang, Yun Seo Jang, Eun-Cheol Park

**Affiliations:** 1 Department of Public Health, Graduate School, Yonsei University, Seoul, Republic of Korea; 2 Institute of Health Services Research, Yonsei University, Seoul, Republic of Korea; 3 Department of Preventive Medicine, Yonsei University College of Medicine, 50 Yonsei-to, Seodaemun-Gu, Seoul, 03722 Republic of Korea

**Keywords:** Work-related physical activity, Depressive symptoms, Patient Health Questionnaire-9, Korean National Health and Nutrition Examination Survey, Leisure

## Abstract

**Background:**

The workplace experiences of employees can impact their mental health. Depressive symptoms, which are experienced by workers, are a mental health issue that deserves attention. Several studies have evaluated physical activity to prevent possible depression in workers in a work environment, however, research on physical activity and depression symptoms directly related to work is still insufficient. Therefore, we aimed to identify the relationship between work-related physical activity and depression among South Korean workers.

**Methods:**

We used data from the Korean National Health and Nutrition Examination Survey conducted in 2014, 2016, 2018, and 2020, which included 31,051 participants. We excluded, participants aged < 15 years (*n* = 4,663), unemployed and economically inactive persons (*n* = 9,793), those who did not engage in work-related physical activities (*n* = 1,513) and leisure physical activities (*n* = 1,558), or those with missing data (*n* = 450). Therefore, the study included 13,074 participants. Work-related activity was measured by self-reporting, while depressive symptoms were measured using the Patient Health Questionnaire-9 (PHQ-9). Multiple logistic regression analysis was performed to investigate the association between work-related physical symptoms and depressive symptoms among workers.

**Results:**

Individuals who engaged in work-related physical activity had higher PHQ-9 scores than those who did not (male: odds ratio [OR]: 1.71, 95% confidence interval [CI]: 1.16–2.52; female: OR: 2.33, 95% CI: 1.66–3.29). High-intensity work-related physical activity significantly increased depressive symptoms (male: OR: 2.15, 95% CI: 1.04–4.43; female: OR: 2.90, 95% CI: 1.46–5.96). When classified according to the severity of depressive symptom, the OR of depressive symptoms of workers engaged in both leisure and work-related physical activities tended to be lower than that of those engaged only in work-related physical activities.

**Conclusion:**

Korean workers who engaged in work-related physical activities exhibited more depressive symptoms. Therefore, our findings suggest that balancing work-related and leisure physical activities can help Korean workers prevent development of depressive symptoms.

**Supplementary Information:**

The online version contains supplementary material available at 10.1186/s12889-023-16631-6.

## Background

As of 2021, wage workers in Korea had an annual working time of 1,915 h, ranking third among the Organization for Economic Co-operation and Development (OECD) countries. Korean workers spent 199 h more at work compared with that spent by other OECD workers [1]. Workplace experiences are closely linked with the mental health of Koreans or modern individuals who spend a significant amount of time at work [2]. Depression and depressive symptoms rank high among the mental disorders experienced by employees [3, 4] and are important, as evidenced by the fact that 7.4% of Korean workers have been diagnosed with depression by medical experts [5]. Depression is accompanied by other symptoms that can reduce an individual’s lifespan and functioning and lead to family and social problems [6]. Therefore, several studies have attempted to prevent and improve depressive symptoms in workers [7].

Physical activity is one of the methods for treating and preventing depression [8]. Numerous studies have reported positive results regarding the relationship between physical activity and mental health [9–13]. Physical activity can help reduce depressive symptoms by increasing the production of endorphins, which are natural mood-enhancing chemicals in the brain. Exercise also promotes better sleep patterns, boosts self-esteem, provides a sense of accomplishment, and helps individuals cope with stress and anxiety [14, 15]. Similarly, studies have indicated that low levels of physical activity are associated with an increased risk of depression[16]. However, some studies have found no association between physical activity and depression and show that work-related physical activity may not be as beneficial and potentially helpful as leisure time physical activity [17, 18]. Furthermore, some studies showed that the impact of physical activity on depression varies depending on the type of physical activity [19, 20].

Most of the existing studies have predominantly focused on the workplace stress and depression [21–24] or the types of physical activity and depressive symptoms. Many previous studies have reported positive results regarding the association between physical activity and mental health. However, when physical activity is disaggregated, not all types of physical activity have consistently shown positive effects. Also, there remains a relative lack of focused research on the association between occupational physical activity and depression among workers. In this study, we aimed to investigate the relationship between work-related depressive symptoms among a representative sample of Korean workers. Additionally, we examined whether there are differences in the intensity of work-related physical activity and the presence of depressive symptoms among subgroups. The results of this subgroup analysis will help determine possible variations in the relationship between the intensity of work-related physical activity and symptoms of depression.

## Methods

### Data

Data were obtained from the Korea National Health and Nutrition Examination Survey (KNHANES) conducted in 2014, 2016, 2018, and 2020. The KNHANES is a nationwide cross-sectional survey that accurately assesses the health and nutritional status of Korean individuals. This survey includes people aged 1 year and older who could represent the Korean population. Through this survey, the Korean Center for Disease Control and Prevention investigates the workers’ health profile, such as presence of obesity, high blood pressure, diabetes, and energy intake; prepares the national health statistic data; and uses these data as a basis for establishing national policies and comparing health levels between countries.

### Participants

This study used the data from the KNHANES conducted in 2014, 2016, 2018, and 2020, which included 31,051 participants. As this study targeted adult workers receiving salaries, participants aged < 15 years (*n* = 4,663), unemployed and economically inactive populations (*n* = 9,793), those who did not engage in work-related physical activities (*n* = 1,513) and leisure physical activities (*n* = 1,558), or those with missing data (*n* = 450) were excluded. Hence, only 13,074 participants (6,804 male and 6,270 female workers) were included in the study. This study did not require prior consent or approval from an Institutional Review Board because the KNHANES data are secondary datasets and consist of de-identified data that are available in the public domain.

### Variables

The main variable of interest in the KNHANES questionnaire was consistent work-related physical activity, including those of medium and high intensity. The participants’ work-related activities were calculated based on their responses to the following questions from the Global Physical Activity Questionnaire (GPAQ) [25]: Does your job involve high-intensity physical activity that causes shortness of breath or increases your heartbeat for at least 10 min? Do you perform moderate-intensity physical activity that makes you slightly out of breath or slightly increases your heartbeat for at least 10 min? According to the KNHANES, “high-intensity activity” is defined as an activity that causes shortness of breath or markedly increases the heartbeat, while “medium-intensity activity” is defined as an activity that causes slight shortness of breath or slightly increases the heartbeat. The participants were further divided into those who engaged in work-related physical activity and those who did not. In addition, the subgroup analysis was classified according to the presence or absence of medium- and high-intensity physical activity.

**Fig. 1:**
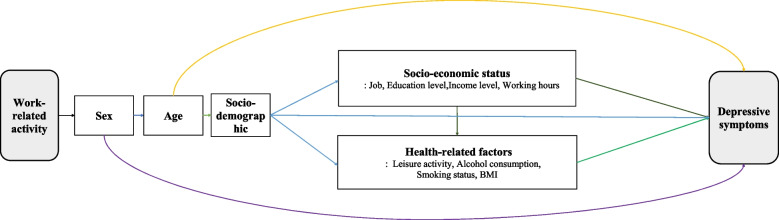
Directed Acyclic Graph representing the relationship between work-related physical activity and depressive symptoms

Depressive symptoms were considered as the dependent variable and evaluated using the Patient Health Questionnaire-9 (PHQ-9). The PHQ-9 consists of nine questions that can assist in diagnosing major depressive disorders. It is a self-administered questionnaire that assesses the patients’ symptoms in the past two weeks, with scores ranging from 0 to 27. This questionnaire has been shown to be a reliable tool for screening for depression and assessing the severity of the illness. Individuals who score higher than 10 on this questionnaire are considered to be at risk for depression [26]. The following independent variables were included in the analysis: sociodemographic factors such as age (19–29, 30–39, 40–49, 50–59, and 60 years or older), region (metropolitan or rural), education level (under middle school, high school, or university), marital status (married or single), job type (white, pink, or blue), average weekly working hours (40 h, 40–52, or 52 h or more), and personal income (low, medium, or high). Health-related factors included alcohol consumption (yes or no), smoking status (yes or no), body mass index (low < 18.5 kg/m2; middle: 18.5–23 kg/m2; high ≥ 23 kg/m2), and level of stress awareness (low, medium, or high). In addition, adjustments were made for other types of physical activity, such as medium- and high-intensity leisure activities (yes or no). The theorized relationship between work-related physical activity and depressive symptoms, and other covariates is represented through a Directed Acyclic Graph (DAG) (Fig. 1). In this DAG, all covariates were considered potential confounders of the association between work-related physical activity and depressive symptoms.

### Statistical analyses

All analyses were stratified by sex due to the differences in physical conditions between male and female [27]. A descriptive analysis was performed using the chi-square test to examine the distribution of the general characteristics of the study population. After considering potentially confounding variables, multiple logistic regression analyses were performed to investigate the association between work-related physical activity and depressive symptoms among workers. Subgroup analyses were performed to investigate the combined effects of each covariate on risk of depression and work-related physical activity. Odds ratios (ORs) and 95% confidence intervals (CIs) were calculated to compare the data of participants with depressive symptoms. The variables were clustered, stratified, and weighted to account for the small number of participants who were retained in the final analysis [28].

## Results

Table 1 presents a summary of the general characteristics of the study population, stratified by sex. Of the 13,074 participants, 6,804 were male and 6,270 were female workers [1]. Of them, 914 male and 522 female workers engaged in work-related physical activity. Overall, incidence rate of depressive symptoms was higher in females than in males (male: 2.6%, female: 5.1%).

**Table 1:** General characteristics of the study population

	**Depressive symptoms (PHQ-9)**													
**Variables**	**Male**						***P-value***	**Female**						***P-value***
	**Total**		**No**		**Yes**			**Total**		**No**		**Yes**		
	**N**	**%**	**N**	**%**	**N**	**%**		**N**	**%**	**N**	**%**	**N**	**%**	
**Total (*N* = 13,074)**	**6,804**	**100.0**	**6,624**	**97.4**	**180**	**2.6**		**6,270**	**100.0**	**5,949**	**94.9**	**321**	**5.1**	
Work- related Physical Activity							< .0001							< .0001
Yes	914	13.4	870	95.2	44	4.8		522	8.3	458	87.7	64	7.0	
No	5,890	86.6	5,754	97.7	136	2.3		5,748	91.7	5,491	95.5	257	4.4	
Leisure Physical Activity							0.001							0.142
Yes	2,502	36.8	2,458	98.2	44	1.8		1,441	23.0	1,378	95.6	63	2.5	
No	4,302	63.2	4,166	96.8	136	3.2		4,829	77.0	4,571	94.7	258	6.0	
Age							< .0001							< .0001
19–29	658	9.7	635	96.5	23	3.5		865	13.8	798	92.3	67	7.7	
30–39	1,396	20.5	1337	95.8	59	4.2		1,071	17.1	1,014	94.7	57	5.3	
40–49	1,561	22.9	1521	97.4	40	2.6		1,422	22.7	1,377	96.8	45	3.2	
50–59	1,526	22.4	1504	98.6	22	1.4		1,479	23.6	1,423	96.2	56	3.8	
60 ≤	1,663	24.4	1627	97.8	36	2.2		1,433	22.9	1,337	93.3	96	6.7	
Region							0.368							0.622
Urban	2,952	43.4	2,868	97.2	84	2.8		2,779	44.3	2,641	95.0	138	5.0	
Rural	3,852	56.6	3,756	97.5	96	2.5		3,491	55.7	3,308	94.8	183	5.2	
Education							0.650							< .0001
Under middle school	1,321	19.4	1,282	97.0	39	3.0		1,742	27.8	1,616	92.8	126	7.2	
High school	2,333	34.3	2,270	97.3	63	2.7		2,087	33.3	1,985	95.1	102	4.9	
University and over	3,150	46.3	3,072	97.5	78	2.5		2,441	38.9	2,348	96.2	93	3.8	
Marriage Status							0.001							0.017
with spouse	5,583	82.1	5,452	97.7	131	2.3		5,121	81.7	4,875	95.2	246	4.8	
single	1,221	17.9	1,172	96.0	49	4.0		1,149	18.3	1,074	93.5	75	6.5	
Job							0.057							0.000
White collar	2,695	39.6	2,635	97.8	60	2.2		2,535	40.4	2,440	96.3	95	3.7	
Pink collar	1,017	14.9	980	96.4	37	3.6		1,884	30.0	1,765	93.7	119	6.3	
Blue collar	3,092	45.4	3,009	97.3	83	2.7		1,851	29.5	1,744	94.2	107	5.8	
Working hours /week							0.003							0.009
low(> 40)	3,112	45.7	3,033	97.5	79	2.5		4,102	65.4	3,913	95.4	189	4.6	
average(41–52)	2,017	29.6	1,978	98.1	39	1.9		1,275	20.3	1,206	94.6	69	5.4	
over(< 52)	1,675	24.6	1,613	96.3	62	3.7		893	14.2	830	92.9	63	7.1	
Income							< .0001							< .0001
Low	1,388	20.4	1,321	95.2	67	4.8		1,347	21.5	1,253	93.0	94	7.0	
Middle	3,620	53.2	3,540	97.8	80	2.2		3,251	51.9	3,066	94.3	185	5.7	
High	1,796	26.4	1,763	98.2	33	1.8		1,672	26.7	1,630	90.8	42	2.5	
BMI^a^							0.001							0.833
Low	131	1.9	121	92.4	10	7.6		332	5.3	313	94.3	19	5.7	
Middle	2,754	40.5	2,678	97.2	76	2.8		3,533	56.3	3,356	95.0	177	5.0	
High	3,919	57.6	3,825	97.6	94	2.4		2,405	38.4	2,280	94.8	125	5.2	
Smoking							< .0001							< .0001
Yes	2,606	38.3	2,503	96.0	103	4.0		362	5.8	302	83.4	60	16.6	
No	4,198	61.7	4,121	98.2	77	1.8		5,908	94.2	5,647	95.6	261	4.4	
Drinking							0.6287							0.626
Yes	6,571	96.6	6,396	97.3	175	2.7		5,504	87.8	5,225	94.9	279	5.1	
No	233	3.4	228	97.9	5	2.1		766	12.2	724	94.5	42	5.5	
Stress Recognition Level							< .0001							< .0001
Low	1,032	15.2	1,029	99.7	3	0.3		815	13.0	810	99.4	5	0.6	
Middle	4,031	59.2	3,992	99.0	39	1.0		3,573	57.0	3,518	98.5	55	1.5	
High	1,741	25.6	1,603	92.1	138	7.9		1,882	30.0	1,621	86.1	261	13.9	
Year							0.094							0.016
2014	1,463	21.5	1,415	96.7	48	3.3		1,370	21.9	1,280	93.4	90	6.6	
2016	1,787	26.3	1,739	97.3	48	36.0		1,597	25.5	1,512	94.7	85	5.3	
2018	1,837	27.0	1,837	100.0		48.0		1,783	28.4	1,698	95.2	85	4.8	
2020	1,634	24.0	1,633	99.9	1	0.1		1,520	24.2	1,459	96.0	61	4.0	

*BMI* Body mass index

^a^Low < 18.5 kg/m2; Middle: 18.5–23 kg/m2; High, ≥ 23 kg/m2

Table 2 shows the association between work-related physical activity and depressive symptoms. Depending on the type of work-related physical activity, both participants showed higher depressive symptoms in the work-related physical activity group than in the non-work-related physical activity group (male: OR: 1.71, 95% CI: 1.16–2.52; female: OR: 2.33, 95% CI: 1.66–3.29). Male participants who engaged in leisure-related physical activity had lower depressive symptoms (OR: 0.60, 95% CI: 0.42–0.87) compared to those who did not, while no significant association was observed between leisure-related physical activity and depressive symptoms in female participants.

**Table 2:** Association between Depression and subject demographic

Variables	Male		Female	
	Depressive symptoms (PHQ-9)		Depressive symptoms (PHQ-9)	
	OR	95% CI	OR	95% CI
Work- related Physical Activity				
No	1.00		1.00	
Yes	1.71	(1.16—2.52)	2.33	(1.66—3.29)
Leisure Physical Activity				
No	1.00		1.00	
Yes	0.60	(0.42—0.87)	1.06	(0.77—1.46)

Table 3 shows the relationship between work-related physical activity and depressive symptoms of workers by sex. For male, the highest odds ratio (OR) was observed among those with a high school education level (OR: 2.57, 95% CI: 1.37–4.83). On the other hand, for female, engaging in work-related physical activity across all educational levels was associated with depressive symptoms, but the highest OR was observed among those with a middle school education or lower (OR: 4.73, 95% CI: 2.30–9.70). Male participants who worked in blue-collar occupations showed the highest incidence of depressive symptoms (male: OR: 1.94, 95% CI: 1.07–3.54; female: OR: 3.00, 95% CI: 1.37–6.55). Additionally, among male and female workers who worked less than the average working time, those who engaged in work-related physical activity tended to show higher incidence of depressive symptoms (male: OR: 2.44, 95% CI: 1.27–4.66; female: OR: 2.77, 95% CI: 1.69–4.55) than those who did not engage in work-related physical activity.

**Table 3:** Results of subgroup analysis stratified by independent variables

	Male			Female		
	Depressive symptoms (PHQ-9)					
	Work- related Physical Activity			Work- related Physical Activity		
	No		Yes	No		Yes
	OR	OR	95% CI	OR	OR	95% CI
Leisure Physical Activity						
No	1.00	1.81	(1.08—3.04)	1.00	2.09	(1.33—3.30)
Yes	1.00	1.72	(0.84—3.53)	1.00	3.67	(1.74—7.75)
Age						
19–29	1.00	2.41	(0.87—6.68)	1.00	1.81	(0.80—4.13)
30–39	1.00	2.18	(1.05—4.53)	1.00	2.78	(1.29—5.98)
40–49	1.00	1.52	(0.63—3.65)	1.00	1.77	(0.60—5.19)
50–59	1.00	1.46	(0.34—6.25)	1.00	1.10	(0.37—3.26)
60 ≤	1.00	1.35	(0.44—4.11)	1.00	9.44	(4.47—19.94)
Region						
Urban	1.00	1.61	(0.88—2.94)	1.00	2.69	(1.39—5.20)
Rural	1.00	2.20	(1.25—3.86)	1.00	2.20	(1.38—3.53)
Education Level						
Under middle school	1.00	2.05	(0.81—5.16)	1.00	4.73	(2.30—9.70)
High school	1.00	2.57	(1.37—4.83)	1.00	1.93	(1.02—3.65)
University and over	1.00	1.33	(0.65—2.72)	1.00	2.19	(1.04—4.59)
Marital state						
Married	1.00	1.86	(1.15—3.03)	1.00	2.39	(1.48—3.87)
Single	1.00	1.70	(0.81—3.57)	1.00	2.56	(1.19—5.52)
Job						
White collar	1.00	1.69	(0.69—4.18)	1.00	1.82	(0.90—3.69)
Pink collar	1.00	1.85	(0.75—4.53)	1.00	2.19	(1.09—4.38)
Blue collar	1.00	1.94	(1.07—3.54)	1.00	3.00	(1.37—6.55)
Working hours /week						
low(> 40)	1.00	2.44	(1.27—4.66)	1.00	2.77	(1.69—4.55)
average(41–52)	1.00	1.37	(0.60—3.13)	1.00	2.24	(1.01—4.96)
over(< 52)	1.00	2.05	(1.03—4.08)	1.00	1.92	(0.74—4.94)
Income						
Low	1.00	1.45	(0.67—3.14)	1.00	5.53	(2.79—10.96)
Middle	1.00	1.27	(0.69—2.35)	1.00	2.06	(1.21—3.53)
High	1.00	6.60	(2.93—14.89)	1.00	0.80	(0.26—2.48)
BMI^a^						
Low	1.00	< 0.001	(< 0.001—0.66)	1.00	4.63	(0.61—35.07)
Middle	1.00	2.53	(1.37—4.67)	1.00	1.92	(1.13—3.26)
High	1.00	1.61	(0.89—2.90)	1.00	2.87	(1.52—5.43)
Smoking						
Yes	1.00	1.93	(1.12—3.33)	1.00	3.92	(1.47—10.44)
No	1.00	1.56	(0.79—3.07)	1.00	2.22	(1.42—3.46)
Drinking						
Yes	1.00	1.79	(1.16—2.77)	1.00	2.33	(1.54—3.53)
No	1.00	1.38	(0.09—20.95)	1.00	3.51	(0.98—12.62)
Stress Recognition Level						
Low	1.00	1.90	(0.04—95.86)	1.00	153.84	(3.10—> 999.99)
Middle	1.00	2.86	(1.35—6.05)	1.00	1.89	(0.77—4.65)
High	1.00	1.59	(0.97—2.61)	1.00	2.38	(1.55—3.65)
Year						
2014	1.00	2.58	(1.24—5.36)	1.00	1.39	(0.73—2.64)
2016	1.00	2.73	(1.07—6.97)	1.00	3.02	(1.32—6.90)
2018	1.00	1.38	(0.43—4.00)	1.00	3.34	(1.57—7.09)
2020	1.00	0.94	(0.39—2.31)	1.00	2.96	(1.28—6.84)

*BMI* Body mass index;

^a^Low < 18.5 kg/m2; Middle: 18.5–23 kg/m2; High, ≥ 23 kg/m2

Tables 4 and 5 shows the results of subgroup analysis stratified by classified depressive symptoms. In Table 4, male who individuals engaged in moderate- and high-intensity job-related tasks had the highest prevalence of moderate depressive symptom (middle OR: 1.94, 95% CI: 1.14–3.30; high OR: 3.04 95% CI: 1.37–6.73). Whereas for female, those involved in any physical activity task level related to their work had the highest prevalence of high-intensity depressive symptoms (middle OR: 6.46, 95% CI: 3.16–13.21; high OR: 7.19 95% CI: 2.35–22.02). In Table 5, Both male and female experienced the highest OR values for severe depressive symptoms when engaged only in work-related physical activities.

**Table 4:** Association between work-related physical activity intensity and each components of depressive symptoms

		Depressive symptoms (PHQ-9, Ref: None ≤ 4)					
Variables		Mild(5–9)		Moderate(10–14)		Severe (15–27)	
		OR	95% CI	OR	95% CI	OR	95% CI
Work- related Physical Activity Intensity							
Male	None	1.00		1.00		1.00	
	Middle	1.57	(1.15–2.14)	1.94	(1.14–3.30)	1.45	(0.47–4.46)
	High	2.26	(1.47–3.46)	3.04	(1.37–6.73)	2.67	(0.55–13.01)
Female	None						
	Middle	1.89	(1.43–2.50)	2.14	(1.25–3.64)	6.46	(3.16–13.21)
	High	2.40	(1.31–4.38)	3.49	(1.49–8.15)	7.19	(2.35–22.02)

**Table 5:** Association between each type of physical exercise and each components of depressive symptoms

		Depressive symptoms (PHQ-9, Ref: None ≤ 4)					
Variables		Mild(5–9)		Moderate(10–14)		Severe (15–27)	
		OR	95% CI	OR	95% CI	OR	95% CI
Type of Work’s Physical Activity							
Male	None	1.00		1.00		1.00	
	Work- related	1.78	(1.26–2.52)	2.08	(1.15–3.76)	2.12	(0.79—5.71)
	Work- related and Leisure	1.39	(0.97–1.99)	1.76	(0.95—3.24)	0.25	(0.03—2.16)
Female	None						
	Work- related	2.21	(1.63–2.98)	2.19	(1.25–3.83)	5.70	(2.65–12.27)
	Work- related and Leisure	1.51	(1.01–2.28)	3.10	(1.40–6.83)	4.19	(1.47–11.95)

**Fig. 2:**
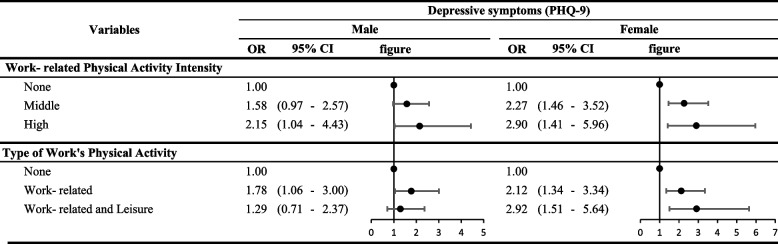
Results of Depressive symptoms and each components of Physical Activity on PHQ-9 scores from 2014,2016,2018 and 2020

Figure 2 illustrates the association between depressive symptoms and intensity of work-related physical activity and types of physical activities. In both male and female workers, higher the intensity of work-related physical activity, higher the incidence of depressive symptoms (male: OR: 2.15, 95% CI: 1.04–4.43; female: OR: 2.90, 95% CI: 1.41–5.96). For male, the OR for moderate-intensity work-related physical activity was not statistically significant. Nonetheless, the results of the specific types of physical activity showed that male workers who engaged in both types of physical activity tended to exhibit lower depressive symptoms compared to male workers who engaged only in work-related physical activity, however, the difference was not statistically significant. In contrast, female workers showed no significant positive change in depressive symptoms based on the type of physical activity.

## Discussion

This study found that after adjusting for potential covariates, Korean workers who engaged in work-related physical activity had an increased risk of developing depressive symptoms than those who did not.

Positive physical activity is an important factor in healthcare and can be a positive factor for improving psychological and physical health [29, 30]. Previous studies have shown that physical activity positively improves the symptoms of depression. Groups that reported frequent activity had low levels of depression: less active groups had higher levels of depression, and inactive groups had the highest levels of depression with semi-linear relationships [29]. Therefore, proper physical activity is essential for maintaining a healthy life [31]. However, the prevalence of sufficient physical activity in Korean workers continues to decrease [32], with office workers in Korea spending longer hours at work [33]. Therefore, they lack sufficient time for engaging in leisure activities [34].

The results of this study differ from those of previous studies as it reports that participation in physical activity had a positive effect on depressive symptoms [35]. Some studies have limited the scope of physical activity to leisure activities and exercise. Previously, physical activity alone was thought to have a positive effect on depressive symptoms as studies were unable to categorize physical activities or equate physical activity with exercise. Workers who engaged in work-related physical activity showed a significant increase in their depressive symptoms. These results suggest that the effects of physical activity on depressive symptoms vary depending on its purpose.

In this study, a subgroup analysis of the independent variables showed that work-related physical activity and depressive symptoms changed according to the education level, working hour, and wage level. Education level can affect an individual's time management. The population with a low level of education spent less time engaging in leisure activities and more time engaging in work-related physical activities than the population with a high level of education [36, 37]. Moreover, depressive symptoms may differ in terms of physical activity and education level [38]. This may explain why depressive symptoms due to physical work activity were higher in the low-education group.

Among male workers, those receiving high wages showed the highest level of depressive symptoms, while female workers receiving low wages showed the highest level of depressive symptoms. In South Korea, female's wages tend to be lower than male's wages [39–41]. This suggests that there may be sex-based differences in the intensity and compensation of work-related physical activity that could explain the variations in depressive symptoms related to work-related physical activity based on different wage levels between sexes.

In both male and female, the likelihood of experiencing depressive symptoms increased as the intensity of work-related physical activity increased. Additionally, when examining depressive symptoms as scores, we observed a tendency for higher depression levels in individuals engaged only in work-related physical activity among both male and female. However, the presence of both work and leisure activities did not show significant associations with depressive symptoms in male. This may indicate that leisure activities have a positive impact on depressive symptoms, aligning with previous findings. People engaged in leisure activities may have a lower likelihood of experiencing depressive symptoms compared to those who do not. This may explain the lack of significance in this aspect for male. However, it should be noted that for females, there may be limitations due to the sensitivity and specificity of the physical activity questionnaire, which could affect the accuracy of leisure activity measurements [42].

Although the results of this study suggest the need to examine the relationship between work-related physical activity and depressive symptoms, it had some limitations. First, because this study used a cross-sectional dataset, only the association could be established; the causal relationship between the variables could not be investigated. Hence, further research is required to confirm this causal relationship. Second, data on work-related and leisure-related physical activities were obtained using a self-reported questionnaire. Therefore, the results may be inaccurate as the participants’ responses were merely based on personal recollection of events. Third, due to limited data, there may be potential risks associated with work-related physical activity and depressive symptoms.

Despite these limitations, this study has several strengths. First, we used standardized tools to measure the work-related physical activity levels and depressive symptoms; therefore, these data can be used as a basis for future research. Second, as this study was conducted on a representative sample, it can be used to reflect the overall situation in Korea and establish better health policies.

## Conclusion

Our findings are important for the overall public health because they provide insights on one possible way to prevent depressive symptoms, a major disease in the modern society, by examining the relationship between work-related physical activity and depressive symptoms among Korean workers. A high-intensity work-related physical activity was related to a high depressive symptom score. Workers who engaged in high-intensity work-related physical activity, those who did not engage in leisure physical activity, and those with low education and income levels were at a higher risk of developing depressive symptoms. These findings suggest that greater the exposure to work-related physical activity, higher the level of depressive symptoms. However, workers who combined work and leisure physical activities showed a lower tendency for depressive symptoms compared to workers who engaged in work-related physical activities alone. Therefore, balancing work-related and leisure physical activity can help Korean workers prevent depressive symptoms. However, further studies are warranted to accurately measure the work-related physical activity level among Korean workers and to clarify the underlying mechanisms of the association between work-related physical activity and depressive symptoms.

### Supplementary Information

**Additional file 1: Supplementary 1. **Association between Depression and subject demographic.

**Additional file 2: Supplementary 2. **Association between Depression and subject demographic.

**Additional file 3: Supplementary 3-1. **Association between Depressive symptoms and subject demographic.

**Additional file 4: Supplementary 3-2. **Results of subgroup analysis stratified by independent variables.

**Additional file 5: Supplementary 3-3. **Association between Depressive symptoms and each components of Work-related Physical Activity.

**Additional file 6: Supplementary 4. **Results of subgroup analysis stratified by independent variables.

## Data Availability

The datasets supporting the conclusions of this article are available in the Korea Centers for Disease Control and Prevention website. (https://knhanes.kdca.go.kr/knhanes/sub01/sub01_01.do).

## Abbreviations

OECD: Organization for Economic Co-operation and Development
KNHANES: Korea National Health and Nutrition Examination Survey
PHQ-9: Patient Health Questionnaire-9
GPAQ: Global Physical Activity Questionnaire
