# Supplementary material for: Association between work-related physical activity and depressive symptoms in Korean workers: data from the Korea national health and nutrition examination survey 2014, 2016, 2018, and 2020
Source: BMC Public Health. 2023 Sep 8;23:1752. doi: 10.1186/s12889-023-16631-6 (PMC10485943; doi:10.1186/s12889-023-16631-6)
Supplement: Supplementary file 1 — Additional file 1: Supplementary 1. Association between Depression and subject demographic. [file 12889_2023_16631_MOESM1_ESM.pdf]

Supplementary1. Association between Depression and subject demographic

Variables	Male		Female	
	Depressive symptoms (PHQ-9)		Depressive symptoms (PHQ-9)	
	OR	95% CI	OR	95% CI
**Work- related Physical Activity**				
No	1.00		1.00	
Yes	1.71	(1.16 - 2.52)	2.33	(1.66 - 3.29)
**Leisure Physical Activity**				
No	1.00		1.00	
Yes	0.60	(0.42 - 0.87)	1.06	(0.77 - 1.46)
**Age**				
19-29	1.00		1.00	
30-39	1.32	(0.74 - 2.38)	0.76	(0.45 - 1.29)
40-49	0.94	(0.49 - 1.79)	0.51	(0.29 - 0.90)
50-59	0.56	(0.27 - 1.17)	0.55	(0.30 - 1.02)
60≤	1.20	(0.57 - 2.53)	1.03	(0.54 - 1.96)
**Region**				
Urban	1.00		1.00	
Rural	0.86	(0.62 - 1.18)	1.09	(0.85 - 1.41)
**Education Level**				
Under middle school	1.55	(0.90 - 2.67)	1.64	(1.04 - 2.61)
High school	1.00	(0.69 - 1.45)	1.11	(0.79 - 1.56)
University and over	1.00		1.00	
**Marital state**				
Married	1.00		1.00	
Single	1.38	(0.89 - 2.14)	0.98	(0.61 - 1.58)
**Job**				
White collar	1.00		1.00	
Pink collar	1.27	(0.80 - 2.01)	1.39	(0.98 - 1.98)
Blue collar	1.12	(0.73 - 1.71)	1.25	(0.84 - 1.85)
**Working hours /week**				
low(>40)	1.71	(1.12 - 2.61)	1.01	(0.74 - 1.39)
average(41-52)	1.00		1.00	
over(<52)	1.56	(1.00 - 2.44)	1.14	(0.76 - 1.72)
**Income**				
Low	1.95	(1.23 - 3.10)	2.23	(1.50 - 3.31)
Middle	1.02	(0.66 - 1.57)	1.95	(1.35 - 2.80)
High	1.00		1.00	
**BMI**[1)]				
Low	1.54	(0.72 - 3.27)	1.02	(0.62 - 1.67)
Middle	1.00		1.00	
High	0.89	(0.64 - 1.23)	1.11	(0.85 - 1.44)
**Smoking**				
Yes	1.52	(1.09 - 2.11)	2.76	(1.91 - 4.00)
No	1.00		1.00	
**Drinking**				
Yes	1.23	(0.49 - 3.09)	0.82	(0.56 - 1.20)
No	1.00	(0.35 - 0.89)	1.00	
**Stress Recognition Level**		(0.54 - 1.33)		
Low	1.00		1.00	
Middle	3.60	(0.81 - 16.04)	3.05	(1.18 - 7.86)
High	31.09	(7.07 - 136.62)	30.09	(11.98 - 75.59)
**Year**				
2014	1.00		1.00	
2016	0.72	(0.46 - 1.12)	0.85	(0.62 - 1.16)
2018	0.54	(0.34 - 0.85)	0.74	(0.52 - 1.05)
2020	0.86	(0.55 - 1.33)	0.62	(0.42 - 0.91)

BMI body mass index;

1) Low <18.5kg/m2; Middle: 18.5–23kg/m2; High, ≥ 23 kg/m2
