# Supplementary material for: Association between work-related physical activity and depressive symptoms in Korean workers: data from the Korea national health and nutrition examination survey 2014, 2016, 2018, and 2020
Source: BMC Public Health. 2023 Sep 8;23:1752. doi: 10.1186/s12889-023-16631-6 (PMC10485943; doi:10.1186/s12889-023-16631-6)
Supplement: Supplementary file 2 — Additional file 2: Supplementary 2. Association between Depression and subject demographic. [file 12889_2023_16631_MOESM2_ESM.pdf]

Supplementary 2. Association between Depression and subject demographic

Variables		Depressive symptoms (PHQ-9)					
		Male			Female		
		β	S.E	P-value	β	S.E	P-value
**Work- related Physical Activity**							
	No	Ref.			Ref.		
	Yes	0.789	0.121	<.0001	1.306	0.206	<.0001
**Leisure Physical Activity**							
	No	Ref.			Ref.		
	Yes	-0.229	0.073	0.002	-0.019	0.099	0.846
**Age**							
	19-29	Ref.			Ref.		
	30-39	0.242	0.169	0.152	-0.067	0.209	0.748
	40-49	0.166	0.175	0.341	-0.483	0.217	0.026
	50-59	0.012	0.178	0.944	-0.342	0.229	0.136
	60≤	0.283	0.187	0.130	-0.068	0.266	0.800
**Region**							
	Urban	Ref.			Ref.		
	Rural	-0.101	0.070	0.151	0.139	0.088	0.116
**Education Level**							
	Under middle school	0.238	0.131	0.068	0.300	0.183	0.102
	High school	0.105	0.087	0.229	0.037	0.116	0.748
	University and over	Ref.			Ref.		
**Marital state**							
	Married	Ref.			Ref.		
	Single	0.633	0.137	<.0001	0.495	0.187	0.008
**Job**							
	White collar	Ref.			Ref.		
	Pink collar	0.085	0.109	0.436	0.329	0.124	0.008
	Blue collar	-0.128	0.094	0.171	0.273	0.138	0.048
**Working hours /week**							
	low(>40)	0.188	0.079	0.018	0.077	0.111	0.488
	average(41-52)	Ref.			Ref.		
	over(<52)	0.197	0.099	0.047	0.213	0.162	0.190
**Income**							
	Low	0.419	0.111	0.000	0.594	0.131	<.0001
	Middle	0.105	0.081	0.195	0.293	0.096	0.002
	High	Ref.			Ref.		
**BMI**[1)]							
	Low	0.463	0.328	0.158	0.219	0.207	0.291
	Middle	Ref.			Ref.		
	High	-0.064	0.076	0.398	-0.062	0.097	0.524
**Smoking**							
	Yes	0.316	0.076	<.0001	1.781	0.274	<.0001
	No	Ref.			Ref.		
**Drinking**							
	Yes	Ref.			Ref.		
	No	0.029	0.184	0.874	-0.235	0.160	0.142
**Stress Recognition Level**							
	Low	Ref.			Ref.		
	Middle	0.728	0.060	<.0001	0.930	0.097	<.0001
	High	2.835	0.106	<.0001	3.690	0.139	<.0001
**Year**							
	2014	Ref.			Ref.		
	2016	-0.222	0.102	0.030	-0.118	0.134	0.377
	2018	-0.328	0.103	0.002	-0.424	0.133	0.001
	2020	-0.180	0.114	0.113	-0.570	0.136	<.0001

BMI body mass index;

1) Low <18.5kg/m2; Middle: 18.5–23kg/m2; High, ≥ 23 kg/m2
