# Supplementary material for: Association between work-related physical activity and depressive symptoms in Korean workers: data from the Korea national health and nutrition examination survey 2014, 2016, 2018, and 2020
Source: BMC Public Health. 2023 Sep 8;23:1752. doi: 10.1186/s12889-023-16631-6 (PMC10485943; doi:10.1186/s12889-023-16631-6)
Supplement: Supplementary file 3 — Additional file 3: Supplementary 3-1. Association between Depressive symptoms and subject demographic. [file 12889_2023_16631_MOESM3_ESM.pdf]

Supplementary 3-1. Association between Depressive symptoms and subject demographic

Variables	Depressive symptoms (PHQ-9)					
	Male			Female		
	β	S.E	P-value	β	S.E	P-value
**Work- related Physical Activity**						
MET < 1	Ref.			Ref.		
MET ≥1	0.789	0.121	<.0001	1.260	0.203	<.0001
**Leisure Physical Activity**						
MET < 1	Ref.			Ref.		
MET ≥1	-0.229	0.073	0.002	-0.016	0.099	0.873
