# Supplementary material for: Association between work-related physical activity and depressive symptoms in Korean workers: data from the Korea national health and nutrition examination survey 2014, 2016, 2018, and 2020
Source: BMC Public Health. 2023 Sep 8;23:1752. doi: 10.1186/s12889-023-16631-6 (PMC10485943; doi:10.1186/s12889-023-16631-6)
Supplement: Supplementary file 4 — Additional file 4: Supplementary 3-2. Results of subgroup analysis stratified by independent variables. [file 12889_2023_16631_MOESM4_ESM.pdf]

Supplementary 3-2. Results of subgroup analysis stratified by independent variables

	Male				Female			
	Depressive symptoms (PHQ-9)							
	Work- related Physical Activity							
	No	Yes			No	Yes		
	β	β	S.E	P-value	β	β	S.E	P-value
**Leisure Physical Activity**								
MET < 1	Ref.	0.880	0.181	<.0001	Ref.	1.331	0.250	<.0001
MET ≥1	Ref.	0.697	0.159	<.0001	Ref.	1.157	0.351	0.001
