# Supplementary material for: Association between work-related physical activity and depressive symptoms in Korean workers: data from the Korea national health and nutrition examination survey 2014, 2016, 2018, and 2020
Source: BMC Public Health. 2023 Sep 8;23:1752. doi: 10.1186/s12889-023-16631-6 (PMC10485943; doi:10.1186/s12889-023-16631-6)
Supplement: Supplementary file 5 — Additional file 5: Supplementary 3-3. Association between Depressive symptoms and each components of Work-related Physical Activity. [file 12889_2023_16631_MOESM5_ESM.pdf]

Supplementary 3-3. Association between Depressive symptoms and each components of Work-related Physical Activity

Variables	Male			Female		
	Depressive symptoms (PHQ-9)			Depressive symptoms (PHQ-9)		
	β	S.E	P-Value	β	S.E	P-Value
**Work- related Physical Activity Intensity**						
MET (= 0)	Ref.			Ref.		
MET (1-700)	0.552	0.180	0.002	1.061	0.332	0.002
MET (>700)	0.852	0.151	<.0001	1.379	0.250	<.0001
**Type of Work's Physical Activity**						
**None**	Ref.			Ref.		
**Work- related**	0.887	0.182	<.0001	1.331	0.253	<.0001
**Work- related and Leisure**	0.485	0.153	0.002	1.052	0.336	0.002
