# Supplementary material for: Association between work-related physical activity and depressive symptoms in Korean workers: data from the Korea national health and nutrition examination survey 2014, 2016, 2018, and 2020
Source: BMC Public Health. 2023 Sep 8;23:1752. doi: 10.1186/s12889-023-16631-6 (PMC10485943; doi:10.1186/s12889-023-16631-6)
Supplement: Supplementary file 6 — Additional file 6: Supplementary 4. Results of subgroup analysis stratified by independent variables. [file 12889_2023_16631_MOESM6_ESM.pdf]

Supplementary4. Results of subgroup analysis stratified by independent variables

	Male				Female			
	Depressive symptoms (PHQ-9)							
	Work- related Physical Activity							
	No	Yes			No	Yes		
	β	β	S.E	P for interaction	β	β	S.E	P for interaction
**Leisure Physical Activity**								
No	Ref.	0.880	0.181	0.236	Ref.	1.331	0.250	0.259
Yes	Ref.	0.697	0.159		Ref.	1.157	0.351	
**Age**								
19-29	Ref.	0.608	0.299		Ref.	0.908	0.385	
30-39	Ref.	0.944	0.240		Ref.	1.727	0.401	
40-49	Ref.	0.711	0.233	0.967	Ref.	0.773	0.342	<.0001
50-59	Ref.	0.841	0.216		Ref.	0.482	0.467	
60≤	Ref.	0.777	0.274		Ref.	3.866	0.799	
**Region**								
Urban	Ref.	0.773	0.192	0.986	Ref.	1.354	0.319	0.746
Rural	Ref.	0.791	0.157		Ref.	1.275	0.260	
**Education**								
Under middle school	Ref.	0.959	0.347		Ref.	2.794	0.598	
High school	Ref.	0.728	0.188	0.637	Ref.	0.822	0.393	<.0001
University and over	Ref.	0.777	0.168		Ref.	1.071	0.230	
**Marital state**								
Married	Ref.	0.860	0.142	0.151	Ref.	1.236	0.237	0.548
Single	Ref.	0.630	0.241		Ref.	1.419	0.407	
**Job**								
White collar	Ref.	0.832	0.219		Ref.	0.907	0.262	
Pink collar	Ref.	0.691	0.288	0.841	Ref.	1.693	0.456	0.025
Blue collar	Ref.	0.798	0.171		Ref.	1.649	0.400	
**Average working hours /week**								
low(>40)	Ref.	0.713	0.187		Ref.	1.202	0.266	
average(41-52)	Ref.	0.572	0.198	0.019	Ref.	1.695	0.392	0.209
over(<52)	Ref.	1.123	0.240		Ref.	1.102	0.576	
**Income**								
Low	Ref.	0.960	0.277		Ref.	1.562	0.485	
Middle	Ref.	0.563	0.140	0.083	Ref.	1.582	0.280	0.007
High	Ref.	1.097	0.299		Ref.	0.436	0.330	
**BMI**								
Low	Ref.	-0.510	0.883		Ref.	1.067	0.658	
Middle	Ref.	1.141	0.214	0.016	Ref.	1.161	0.250	0.305
High	Ref.	0.595	0.144		Ref.	1.713	0.393	
**Smoking**								
Yes	Ref.	0.710	0.139	0.315	Ref.	1.107	0.189	<.0001
No	Ref.	0.871	0.209		Ref.	3.535	1.256	
**Drinking**								
Yes	Ref.	0.976	0.709	0.586	Ref.	1.558	0.612	0.719
No	Ref.	0.791	0.124		Ref.	1.288	0.218	
**Stress Recognition Level**								
Low	Ref.	0.190	0.165		Ref.	0.523	0.652	
Middle	Ref.	0.677	0.130	0.001	Ref.	0.555	0.188	<.0001
High	Ref.	1.247	0.305		Ref.	2.300	0.395	
**Year**								
2014	Ref.	0.917	0.222		Ref.	0.700	0.277	
2016	Ref.	0.875	0.284	0.553	Ref.	1.887	0.481	0.000
2018	Ref.	0.804	0.223		Ref.	2.060	0.506	
2020	Ref.	0.611	0.242		Ref.	1.139	0.446	

BMI body mass index;

1) Low <18.5kg/m2; Middle: 18.5–23kg/m2; High, ≥ 23 kg/m2
